# At the moment of occurrence of a fragility hip fracture, men have higher mechanical properties values in comparison with women

**DOI:** 10.1186/1471-2474-14-295

**Published:** 2013-10-16

**Authors:** Ana C Vale, Inês P Aleixo, Miguel Lúcio, André Saraiva, Joana Caetano-Lopes, Ana Rodrigues, Pedro M Amaral, Luís G Rosa, Jacinto Monteiro, João E Fonseca, Maria F Vaz, Helena Canhão

**Affiliations:** 1 Rheumatology Research Unit, Instituto de Medicina Molecular, Faculdade de Medicina da Universidade de Lisboa, Lisbon, Portugal; 2 Instituto de Ciência e Engenharia de Materiais e Superfícies, Instituto Superior Técnico, University of Lisbon, Av. Rovisco Pais, Lisbon, Portugal; 3 Serviço de Reumatologia e Doenças Ósseas Metabólicas, Lisbon Academic Medical Centre, Lisbon, Portugal; 4 Departamento de Engenharia Mecânica, Instituto Superior Técnico, UL, Av. Rovisco Pais, Lisbon, Portugal; 5 Serviço de Ortopedia, Hospital de Santa Maria, Lisbon, Portugal

**Keywords:** Trabecular bone, Osteoporosis, Fragility fracture, Compression, Mechanical properties

## Abstract

**Background:**

It is well established that males have lower fracture risk in comparison with females, which suggests a higher bone resistance in men. The aim of our study was to find out if in older patients with hip fragility fractures, gender has also an impact on trabecular bone material behaviour, specifically to determine whether trabecular mechanical properties under compressive loading differ between men and women who suffered a fragility hip fracture.

**Methods:**

Femoral epiphyses were consecutively collected during hip replacement surgery due to proximal femur fragility fracture. Trabecular bone cylinders were drilled and submitted to uniaxial compression tests and mechanical properties were assessed.

**Results:**

Seventy-three patients, 55 women (mean age 81 years and standard deviation of 7 years) and 18 men (mean age 81 years and standard deviation of 8 years) were evaluated. The ultimate stress of trabecular bone was significantly higher in men than in women: the median values and the interquartile range (IQR) were respectively 8.04(5.35-10.90) MPa vs. 4.46(3.02-7.73) MPa, (*p-value* = 0.005). The same difference between male and female was observed in the Young’s modulus: 293.68(166.67-538.18) MPa vs. 174.26(73.07-322.28) MPa, (*p-value* = 0.028), and also in the energy to failure: 0.25(0.07-0.42) MJ/m^3^ vs. 0.11(0.05-0.25) MJ/m^3^, (*p-value* = 0.058). These differences were also verified after adjusting the analysis for age in a multivariate model analysis.

**Conclusions:**

Our observations demonstrated that, even in a population who suffered a fragility hip fracture, men still have higher trabecular bone mechanical properties in comparison with women.

## Background

Osteoporosis is a systemic skeletal disease characterized by low bone mass, microarchitectural deterioration and strength impairment, which increase the risk of fragility fractures, leading to high morbidity and reducing patient’s quality of life [1-7]. Bone loss is clinically evaluated by bone mineral density (BMD), but low BMD only explains a minority of the fractures that occur [2,8-10], since bone structure, microarchitecture and material properties also account for bone fragility.

Gender and age influence the risk of fracture [2,11-19]. The peak number of hip fractures occurs at 75–79 years of age for both sexes [11]. However, the hip fracture risk increases in women after 40 years and in male, increases only after 65 years [11]. Moreover, about 70% of all hip fractures occur in women [20]. This might be explained by several factors. On one hand, changes in bone structure and geometry induced by aging contribute to decreased bone strength and increased fragility fracture risk in the elderly population [12,13,16,18,21,22]. On the other hand, important determinants of bone strength are clearly different between genders. In fact, when peak bone mass is achieved, bone density in men is one fourth to one third greater than in women [15] and male bones reach a larger diameter and cortical thickness than female ones [15,18]. Additionally, the pattern of bone loss is different between genders [15]. Bone mass rapidly decreases in women at menopause, around 50 years-old, [18] in contrast with men of the same age. Consequently, differences between genders get more pronounced with aging [12-19]. Another gender difference is related to bone loss at a microstructural level, which occurs mainly by trabecular thinning and reduced bone formation in men and mainly by loss of connectivity between trabeculae in women [1,15,18].

Beyond age and sex, the mechanical properties of bone depend on several factors, such as density and the distribution of bone mass [23-27], geometry [23,27], microarchitecture [28,29], bone composition [8,18,24,30-33], anatomical location [34-36] and concomitant diseases [28,31,32,37-39].

As most fractures at hip, vertebrae or wrist tend to start in the trabecular (cancellous) bone, with a decrease of bone mass and microarchitectural changes, an understanding of the mechanical properties of trabecular bone is extremely important in the evaluation of the risk of fracture [40,41]. Results of tensile and bending tests performed in young, middle-aged, and elderly patients showed that aging significantly decreases cancellous bone strength [42]. However, to our knowledge, there are no studies with compressive tests on trabecular bone that had investigated the effect of both gender and age, performed in an elderly population who had suffered a hip fragility fracture. It is unknown whether trabecular bone of patients who suffered a fragility fracture differs between women and men.

Moreover, given the differences in bone behaviour observed between men and women in other settings, we hypothesized that elderly men who suffered a fragility fracture have different mechanical properties than women. Thus, the aim of this study was to assess and compare the trabecular bone compression behaviour in elderly men and women who suffered a hip fragility fracture.

## Methods

### Patients

Patients who suffered a low-energy hip fracture and underwent total hip replacement surgery at the Orthopedic Department of Hospital de Santa Maria were consecutively recruited for this study from 2007 up to 2009. Demographic and clinical data such as age, gender and surgery reason were collected. Patients with other metabolic bone diseases and bone metastases were excluded. Seventy-three femoral epiphyses from patients submitted to total hip replacement surgery (fifty-five women and eighteen men) due to low-energy fracture were consecutively collected. The mean age of both genders was 81 years-old, where the age range for females from 59 to 96 years-old (standard deviation of 7 years) and for males from 64 to 84 years-old (standard deviation of 8 years). Three age-groups were defined: below 75 years-old (8 females, 4 males), 75 to 85 years-old (29 females, 11 males) and over 85 years-old (18 females, 3 males).

Written informed consent was obtained from all patients and the study was conducted in accordance with the regulations governing clinical trials, such as the Declaration of Helsinki, as amended in Seoul (2008), and was approved by the Ethical Committee of the Lisbon Academic Medical Centre, Portugal.

### Specimen preparation

After the surgical procedure, the femoral epiphyses were immediately stored at -80°C. Before testing, this material was defrosted at room temperature.

We used a perforating drill with a diameter of 15 mm and a length: diameter ratio of 2, with a corresponding final specimen length of 30 mm. The trabecular bone cylinders were obtained by drilling in the highest in vivo loading direction, in accordance with Sun et al. [38]. The cortical shell was cut off.

The cylinders’ ends were polished with an 800 grade silicon carbide paper under water flow (Surface Polishing Machine Struers DAP-V) to make them parallel. Bone cylinders were de-fatted for three hours using a chloroform and methanol solution (1:1 ratio) and were hydrated overnight in phosphate-buffered saline (PBS) solution.

### Compression tests

Uniaxial compression tests were performed in a universal testing machine (model 5566, Instron Corporation, Canton, USA), with a load cell of 10kN and a cross-head rate of 0.1 mm/s that was chosen in accordance with Li and Aspen [31]. All samples were loaded in the principal stress direction (superior-inferior direction). The testing machine was operated by materials testing software (Bluehill2, Instron Corporation, Canton, USA).

Stress–strain (σ-ϵ) curves were obtained for each specimen from the load (F) vs. displacement (ΔL) data acquired, taking into consideration the dimensions of the specimens, area (A) and length (L), with diameter and height measured three times. The stress, σ, is defined as the load divided by the area, σ = F/A, while the strain, ϵ, is the ratio ϵ = ΔL/L. Three mechanical parameters were obtained from the stress–strain curve: Young’s modulus, E (calculated as the slope of the stress–strain curve in the linear elastic region), ultimate stress, σ_ULT_ (the maximum stress that the bone can support without failing), and energy to failure, W_ULT_ (measured by the area under the stress–strain curve until the ultimate stress). The mechanical parameters Young’s modulus, ultimate stress and the energy to failure, were used to evaluate mechanical properties, respectively stiffness, mechanical strength and toughness.

### Statistical analysis

Statistical analysis was performed using a statistical software (version 9.2, SAS Institute Inc., Cary, NC, USA). The Shapiro Wilk test indicated that the continuous outcome variables (Young’s modulus, ultimate stress and energy to failure) had non-normal distributions. Therefore, non-parametric tests were used for statistical analysis and the data were presented as median and interquartile range (Q1-Q3). More specifically, the non-parametric tests, Mann–Whitney and the Kruskal-Wallis tests, were performed to assess comparisons between two (female and male population) and three groups (the three age-groups defined), respectively.

Firstly, with the entire sample, a univariate comparison between female and male was made for each property, using the Mann-Whitney test. In addition, a univariate comparison for determination of age-group differences for each material parameter, using the Kruskal-Wallis test, was done for the entire population. Also, the univariate correlation, given by the Spearman’s correlation coefficient of age as a continuous variable for each material measure, was performed for males and females separately.

Finally, the contribution of the independent variables (gender and age-groups, and gender and age as a continuous variable) on the prediction of each bone mechanical property was determined by two multivariate quartile (median) regression models. Quartile (median) regression consists of a newly developed transformed multivariate linear regression analysis for non-normal outcomes (SAS software, version 9.2).

Differences were considered statistically significant between groups for two-sided *p-value* lower than 0.05.

## Results

**Figure 1:**
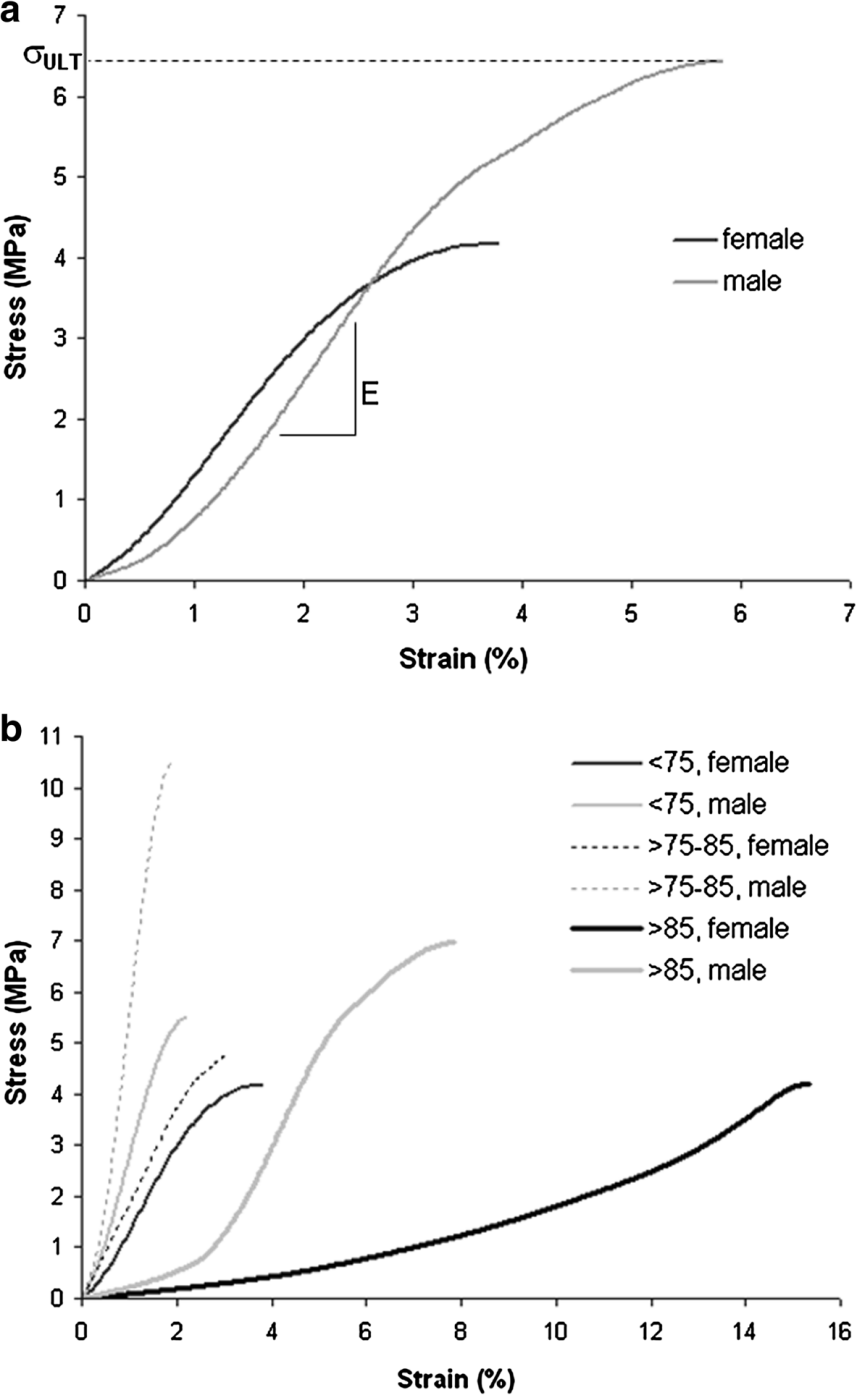
**Experimental compressive stress–strain curves. a)** from female and male fracture: the parameters ultimate stress, σ_ULT_, Young’s modulus, E, are indicated. The energy to failure, W_ULT_, corresponds to the area below the stress–strain curve. **b)** six stress–strain curves for male and female belonging to the three considered age-groups. Each curve represented corresponds to the experimental curve with ultimate stress value closest to the median value corresponding to each age-group.

A typical stress–strain curve from female and male patients is exemplified in Figure 1a), where the determination of important parameters, E, σ_ULT_ and W_ULT_ are indicated. Figure 1b) exhibits six stress–strain curves, for male and female, belonging to the three age-groups considered.

For the overall sample data, when comparing female, F, and male, M, (Table 1), significant differences between genders were detected in bone material parameters (E, σ_ULT_ and W_ULT_). The ultimate stress that evaluates the mechanical strength was the mechanical parameter that exhibited more significant differences (p = 0.005). The Young’s modulus, which is related to stiffness, also presented significant differences between male and female (p = 0.028). In contrast, the energy to failure (i.e. a measure of the toughness) was not significantly different (p = 0.058), but male values tended to be higher than female ones.

**Table 1:** Summary of mechanical parameters by gender: median (IQR)

***Measure***	**Female (*N*** **= 55)**	**Male (*N*** **= 18)**	***p-value***^***a***^
***Age*** [years]	82 (77–86)	81 (78–84)	0.605
***Ultimate stress, *σ**_**ULT**_ [MPa]	4.46 (3.02–7.73)	8.04 (5.35–10.90)	0.005
***Young’s modulus, *E** [MPa]	174.26 (73.07–322.28)	293.68 (166.67–538.18)	0.028
***Energy to failure, *W**_**ULT**_ [MJ/m^3^]	0.11 (0.05–0.25)	0.25 (0.07–0.42)	0.058

Note: IQR = Interquartile range.

^*a*^*p-value* obtained by Mann–Whitney test for univariate comparison between female and male, with statistical significance for *p* < 0.05.

The Young’s modulus, E, and the energy to failure, W_ULT_, exhibited statistically significant differences on age-group comparison, regardless of gender (Table 2). However, only W_ULT_ increased continuously with aging. The average ultimate stress did not change significantly with age, while the Young’s modulus was lower in the oldest group.

**Table 2:** Summary of mechanical parameters by age-group: median (IQR)

***Measure***	**<75 (*N*** **= 12)**	**≥75–85 > (*N*** **= 40)**	**≥ 85 (*N*** **= 21)**	***p-value***^***a***^
***Age*** [years]	70 (66–71)	81 (78–83)	88 (86–93)	<.0001
***Ultimate stress, *σ**_**ULT**_ [MPa]	4.49 (3.85–5.34)	7.03 (3.45–10.99)	5.30 (3.69–6.96)	0.163
***Young’s modulus, *E** [MPa]	238.23 (129.77–328.50)	244.98 (115.96–516.13)	87.72 (29.76–251.70)	0.037
***Energy to failure, *W**_**ULT**_ [MJ/m^3^]	0.06 (0.06–0.09)	0.16 (0.05–0.27)	0.249 (0.10–0.40)	0.037

^*a*^*p-value* obtained by Kruskal-Wallis test for univariate comparison between age-groups, with statistical significance for *p <* 0.05.

**Figure 2:**
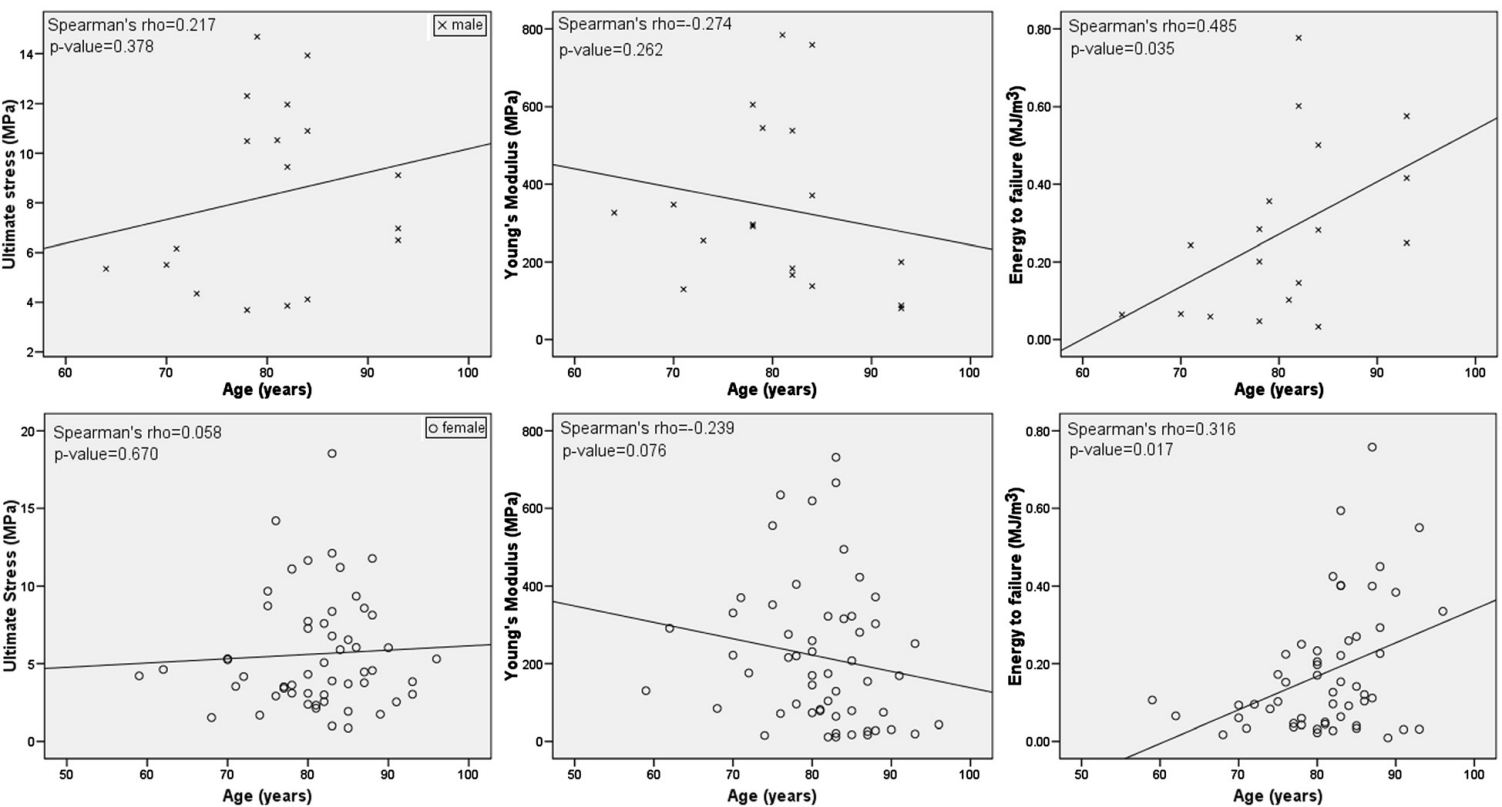
**Mechanical parameters versus age with the respective Spearman’s correlation coefficient (ρ) and p-value, for males and females.** Statistical significance for p < 0.05.

Using age as a continuous variable (Figure 2), again like for age-group analysis, the energy to failure, W_ULT_, was the mechanical parameter that exhibited the highest and the most significant Spearman’s correlation coefficient (ρ) in association with age for both genders (for males, ρ = 0.485, *p-value* = 0.035, and for females, ρ = 0.316, *p-value* = 0.017). Moreover, the energy to failure increases with aging, for both genders, while the ultimate stress, presents a positive Spearman’s correlation coefficient, but with no statistical significance. In contrast, the Young’s modulus decreases with age, but no statistical significance was found.

Two multivariate (bivariate) quartile regression analysis models were performed taking each bone mechanical parameter as the outcome (Table 3): one model had age (as a continuous variable) and gender as covariates, while the other one considered gender and age-group (as a categorical variable) as covariates. This bivariate median regression analysis showed significant differences only in energy to failure, for models that combined age-group and gender (*p-value* of 0.02 and 0.04, respectively). Age was also a significant predictor of energy to failure, since with the first bivariate model, with age and gender, the energy to failure presented a beta coefficient with a significant difference (p = 0.01). All the other parameters, E and σ_ULT_, showed no significant differences for both bivariate median regression models, which suggests that age and gender effects can be confounded in Young’s modulus and ultimate stress prediction by the bivariate models used.

**Table 3:** Multiple median regression analysis for bone material properties

***Variables***	**Age**			**Gender**		
***Measure***	β^a^	**95% Confidence Interval**	*p-*value^*b*^	β^a^	**95% Confidence Interval**	*p-*value^*b*^
***Ultimate stress, *σ**_**ULT**_ [MPa]	0.02	-0.11 < **β** < 0.16	0.72	-2.29	-5.43 < **β** < 0.86	0.15
***Young’s modulus, *E** [MPa]	-6.10	-12.54 < **β** < 0.34	0.06	-97.72	-216.06 < **β** < 20.62	0.10
***Energy to failure, *W**_**ULT**_ [MJ/m^3^]	0.01	0.00 < **β** < 0.02	0.01	-0.09	-0.22 < **β** < 0.03	0.13
***Variables***	**Age-group**			**Gender**		
***Ultimate stress, *σ**_**ULT**_ [MPa]	0.57	-0.68 < **β** < 1.81	0.37	-2.00	-5.27 < **β** < 1.27	0.23
***Young’s modulus, *E** [MPa]	-34.89	-124.20 < **β** < 54.42	0.44	-104.49	-238.15 < **β** < 29.17	0.12
***Energy to failure, *W**_**ULT**_ [MJ/m^3^]	0.09	0.02 < **β** < 0.16	0.02	-0.13	-0.25 < **β** < -0.01	0.04

^***a***^Coefficients obtained by QUANTREG procedure.

^***b***^Statistical significance *p* < 0.05.

Note: Two multiple median regression models were performed. In the first model, each bone material property (the dependent variable) was analysed with age and gender as covariate variables. In the second model, these bone material parameters were analysed with age-group and gender as covariate variables.

## Discussion

Our results showed a significant difference in bone mechanical strength, estimated by the ultimate stress, between elderly men and women (mean age of 81 years-old for both genders) who had experienced a hip fragility fracture.

The Young’s modulus, the ultimate stress and the energy to failure were higher in elderly men than in women, which is in accordance with previous studies that qualitatively predicted lower material properties in women when compared to men [22]. Also one could speculate that male hip fractures occur at higher energy levels [11]. The values obtained in the present work for the mechanical parameters are in agreement with other figures previously published, both in terms of absolute and relative values [31]. The only exception was observed in energy to failure that was slightly lower in male patients as compared to females in the younger age group (less than 75 years-old). Li and Aspen [31] found for the Young’s modulus of the osteoporotic bone E = 247 MPa, while Wang et al. [42] found that average values of the Young’s modulus, obtained by bending tests in different age-groups, decreased with aging and showed values of 209, 207 and 143 MPa, respectively for young, middle age and elderly patients.

Regarding age effects, a decrease in the Young’s modulus and an increase in the energy to failure were detected, while the ultimate stress was almost constant. These results are unexpected, as for example, Wang et al. [42] report that age-dependent changes are reflected in a decreased strength, elastic modulus and work to fracture. However, our age interval is considerably narrow which may have biased that effect.

For the three defined age-groups similar gender differences on each parameter determined were noted and this fact might reveal that the age-related bone degradation can affect both genders in the same way. However, for the oldest age-group, gender-related differences were not detected, probably because, in this context, the gender-effect might be overcame by the impact of age.

There are some limitations in this study that should be taken into account. Firstly, conclusions are only valid for trabecular bone. However, most of the fractures start to occur at the trabecular zones of bones [28,37,43] and thus trabecular fragility can be regarded as a strong suggestive indication of overall bone fragility. In addition to this limitation, there was an imbalance in the proportion of females and males within each age-group, particularly in the case of the general age-group comparison. The reason for this fact is that study’s population includes consecutive human samples obtained from hip replacement surgeries due to hip fracture, a condition that occurs late in life, the age range of the patients was narrow and skewed to older age. Additionally, because this is a condition that occurs more frequently in women, the number of females was also higher. Therefore, the conclusions obtained in the present work have limitations in their extrapolation out of this specific population.

There are several bone microstructural aspects to help to understand the biomechanical results obtained in this study. The microstructure of trabecular bone exhibit age- and gender-related variations [44-46]. Micro-CT may be used to evaluate differences of trabecular microstructure, for example between several age groups [45] at different bone regions. The bone volume fraction decreases with age for both women and men [44,45]. However, for the same age group, the bone volume fraction is higher in men when compared to women [44]. The trabecular thickness also decreases with aging [46]. Besides age and gender, the disease also affects the properties of a single trabeculae. For example, osteoporotic trabeculae showed decreases in Young’s modulus, yield strength and work to failure [47].

Being bone a nanocomposite material consisting of mineral crystals and organic phase, bone strength also depends on its intrinsic material properties [43,48,49]. In fact, elderly women displayed large mineral grains, while small grains indicate the presence of younger bone with a more recently remodelled structure [49]. On the other hand, it is believed that the organic phase contributes to age differences in elasticity, explaining a lower elastic behaviour of elderly bone trabeculae [48]. It was also found that in young trabeculae, the arrangement of the matrix provide an increase of the interface available for cracking between the mineralized fibrils, which may reduce crack propagation and will help to dissipate a large amount of energy [49].

Studies that combine mechanical and microstructural tests showed that there are different relationships between mechanics and structure [28,50]. For osteoporotic samples a decrease on the bone volume fraction is followed by a decrease in the elastic modulus and mechanical strength [50]. It is also known that lower bone volume fraction, lower trabecular number and decreased connectivity are important determinants of hip fracture [28].

## Conclusions

This study reported gender differences in the mechanical properties of trabecular femoral bone of elderly patients who suffered a hip fragility fracture. Based on this population, male trabecular bone showed higher mechanical properties: a higher value of Young’s modulus and ultimate stress when compared to women. Furthermore, the deterioration of bone properties affected more dramatically the Young’s modulus, especially in the oldest patients.

## Competing interests

The authors declare that they have no competing interests.

## Authors’ contributions

ACV acquired and interpreted the experimental data, performed the statistical analysis and drafted the manuscript. IPA, ML and AS made essential contributions for sample preparation and made the experimental tests. JCL and AR participated in sample collection and selected the most suitable samples. JM was the surgeon responsible for the hip replacement surgeries. PMA and LGR contributed for the optimization of the experimental methodology. JEF made substantial contributions in sample selection, in data analysis, drafting and revising the article for important intellectual content. MFV made essential contributions in design of the experimental methodology, in data interpretation, and in drafting the manuscript and in revising. HC made substantial contributions for interpretation of data and in statistical analysis, and was involved in revising the manuscript critically. All authors read and approved the final manuscript.

## Pre-publication history

The pre-publication history for this paper can be accessed here:

http://www.biomedcentral.com/1471-2474/14/295/prepub
